# A longitudinal study of C1q and anti-C1q autoantibodies in homologous and heterologous pregnancies for predicting pre-eclampsia

**DOI:** 10.3389/fimmu.2022.1037191

**Published:** 2022-11-09

**Authors:** Chiara Agostinis, Gabriella Zito, Miriam Toffoli, Isabel Peterlunger, Livia Simoni, Andrea Balduit, Erica Curtolo, Alessandro Mangogna, Beatrice Belmonte, Davide Vacca, Federico Romano, Tamara Stampalija, Tiziana Salviato, Federica Defendi, Nicoletta Di Simone, Uday Kishore, Giuseppe Ricci, Roberta Bulla

**Affiliations:** ^1^Institute for Maternal and Child Health, Istituto di Ricovero e Cura a Carattere Scientifico (IRCCS), Burlo Garofolo, Trieste, Italy; ^2^Department of Medical, Surgical and Health Science, University of Trieste, Trieste, Italy; ^3^Tumor Immunology Unit, Department of Health Sciences, University of Palermo, Palermo, Italy; ^4^Institute of Pathology, University of Modena and Reggio Emilia, Modena, Italy; ^5^Laboratory of Immunology, Institute of Biology and Pathology, Centre Hospitalier Universitaire Grenoble Alpes, La Tronche, France; ^6^Department of Biomedical Sciences, Humanitas University, Milan, Italy; ^7^Istituto di Ricovero e Cura a Carattere Scientifico (IRCCS) Humanitas Research Hospital, Milan, Italy; ^8^Department of Veterinary Medicine, United Arab Emirates University, Al Ain, United Arab Emirates; ^9^Department of Life Sciences, University of Trieste, Trieste, Italy

**Keywords:** C1q, anti-C1q autoantibodies, ART pregnancy, pre-eclampsia, oocyte donation

## Abstract

C1q, the recognition molecule of the classical pathway of the complement system, plays a central role in pregnancy. Lack of C1q is characterized by poor trophoblast invasion and pregnancy failure. C1q can be the target of an antibody response: anti‐C1q autoantibodies (anti-C1q) are present in several infectious and autoimmune diseases. The presence of these autoantibodies has been detected also in 2-8% of the general population. Recent evidence indicates that women who undergo assisted reproductive technology (ART) have an increased risk of developing pre-eclampsia (PE), particularly oocyte donation (OD) pregnancies. The aim of this study was to characterize the levels of C1q and anti-C1q in PE gestations, in healthy spontaneous, homologous and heterologous ART pregnancies. Serum of the following four groups of women, who were followed throughout two or three trimesters, were collected: PE, patients diagnosed with PE; OD, oocyte donation recipients; HOM, homologous ART women; Sp, spontaneous physiological pregnancy. Our results indicate that PE patients have lower levels of anti-C1q. In ART pregnant women, the trend of C1q and anti-C1q levels were similar to PE patients, even though these women did not develop PE-like symptoms during pregnancy. This finding suggests an immunological dysfunction at the foetal-maternal interface in ART pregnancies, a hypothesis confirmed by the observation of C1q deposition in placentae derived from OD, comparable to PE. Since significantly lower levels of anti-C1q were detected in PE compared to healthy control sera, we hypothesize the possible binding on placental syncytiotrophoblast microvesicles (STBM), which are increased in the circulation of PE mothers. Furthermore, the characterization of the binding-epitope of anti-C1q revealed that “physiological” autoantibodies were mainly directed against C1q globular domain. We concluded that anti-C1q could have a physiological role in pregnancy: during the healthy spontaneous pregnancy the raised levels of these autoantibodies can be important for the clearance of STBM. In PE and in pathological pregnancies (but also in OD pregnancies), the increase in syncytiotrophoblast apoptosis and consequent increase of the circulating STMB levels lead to a consumption of C1q and anti-C1q.

## Introduction

Pre-eclampsia (PE) is a gestation-related disorder characterized by the onset of hypertension, proteinuria or organ dysfunction in the second half of pregnancy (1). It is responsible for severe morbidities involving both mothers and newborns, and in rare cases, can cause even death. The worldwide prevalence of PE has been reported to be around 4.6% (2). Interestingly, an increased risk of PE (OR: 2.94, 95%CI: 2.27-7) has been observed in assisted reproductive technology (ART) patients, especially in patients receiving oocyte donation (OD), as compared to naturally conceived pregnancies (3). Since the first successful OD pregnancy in 1984, the number of reported treatments in Europe has registered a continuous increase (4, 5) due to the progressive amelioration of ART techniques. Thus, the increased risk of PE in these patients merits better understanding of the potential causes of PE in order to identify early predictive biomarkers for early diagnosis, preventive treatments and a more efficient patient management, especially in heterologous and homologous pregnancies.

The exact pathophysiology of PE has not been fully clarified yet; placental vascular dysfunction is considered its main cause due to ineffective trophoblast invasion and arterial remodeling (6). On the foetal side, PE can cause reduced blood flow and subsequently intrauterine foetal growth restriction (IUGR) and/or preterm birth. On the maternal side, the pathologic placenta releases apoptotic/necrotic debris and antiangiogenic factors in the maternal circulation, thus inducing endothelial dysfunction and a general inflammatory reaction (7, 8). This condition can result in a heterogenous clinical condition characterized by hypertension, renal insufficiency, liver involvement, and hematologic and neurological complications. Despite the absence of a general consensus about the initial trigger, a wide range of immunological, angiogenic, and genetic factors have been demonstrated as contributors to the development of PE (9).

A key element involved in the PE pathogenesis is an immune imbalance at the foetal-maternal interface. As the fetus also inherits half of paternal antigens, the establishment of the immunological tolerance during pregnancy is essential to prevent the rejection of the semi-allogeneic fetus by the maternal immune system (10). In ART, the impairment of tolerance mechanisms seems responsible for higher prevalence of PE, especially in heterologous pregnancies, the fetus being completely allogenic to the mother (11).

C1q, the first recognition molecule of the classical pathway of complement system (C), represents a double-edged molecule in determining pregnancy outcomes. Being essential for the correct placentation, C1q is synthesized by extravillous trophoblasts to promote the interstitial migration and to act as a molecular bridge between endovascular trophoblast and maternal endothelial cells of the spiral arteries (12, 13). In animal models, C1q deficiency caused the development of a dysfunctional placenta and PE-like symptoms (14). A recent paper has considered C1q as a biomarker of extravillous trophoblast contributing to decidual vascular remodeling (15). Conversely, lower levels of C components were detected in the sera of PE patients due to C consumption and increased deposition of activated C components in the placenta (16). Circulating C1q level has been shown to diminish in PE women, probably due to its binding to placental apoptotic bodies, syncytiotrophoblast microvesicles (STBM) and debris which are increased in the circulation of PE patients (17).

Anti-C1q autoantibodies (anti-C1q) are often detected in patients with autoimmune and infectious diseases (18, 19). Although their biological functions remain far from clear, anti-C1q have been recently considered to play a role in adverse pregnancy outcomes (20, 21); however, no evidence of the involvement of anti-C1q in PE has been reported.

In this study, we have examined the presence and the specificity of anti-C1q in PE patients and in healthy pregnancies. We have also evaluated the involvement of C1q and anti-C1q in ART pregnancies, as they are considered at a higher risk for developing the disease, aiming to find early predictive markers for PE in OD pregnancies. Understanding the immunological changes in OD pregnancies will be a first step towards preventative strategies and potential preconception screenings in infertile women.

## Material and methods

### Patients

In the current study, we analyzed three different cohorts of patients, as summarized in **Supplementary Table 1**. The first cohort was enrolled at the Institute for Maternal and Child Health of the “Policlinico Gemelli” (Rome, Italy): serum samples were collected from 30 PE patients, which were matched for age, parity, and gestational age to 30 healthy pregnant women (CTRL), at PE diagnosis (second/third trimester of pregnancy; **Supplementary Table 2**). The cohort of pregnant women for prospective studies was enrolled at the Prenatal Diagnosis and Gynaecologic Unit of the Institute for Maternal and Child Health, IRCCS “Burlo Garofolo” (Trieste, Italy): we collected the sera from first trimester pregnancy of 20 PE patients, matched with CTRL for maternal age (32 ± 4) and parity. The last cohort included those attending the Physiopathology of Human Reproduction and Medically Assisted Procreation Clinic of IRCSS “Burlo Garofolo” (Trieste, Italy). Four groups of pregnant women were enrolled in this cohort: I) OD pregnancies, and II) homologous *in vitro* fertilization (IVF) pregnancies (HOM), both enrolled at the Physiopathology of Human Reproduction and Medically Assisted Procreation Clinic of IRCSS “Burlo Garofolo” (Trieste, Italy); III) spontaneous pregnancies (Sp CTRL) from the Obstetric and Gynaecological Clinic of IRCCS “Burlo Garofolo” (Trieste, Italy); and IV) pregnant women diagnosed with PE at the Obstetric and Gynaecological Clinic of IRCCS “Burlo Garofolo” (Trieste, Italy) or at the Institute for Maternal and Child Health of “Policlinico Gemelli” (Rome, Italy). The main characteristics of ART patients (OD and HOM) and Sp CTRL are summarized in **Table 1**. They did not develop PE-like clinical symptoms during pregnancy. Comparing the three groups, the variables included: mean age in years, parity, Body Mass Index (BMI), delivery outcomes, pregnancy complications, pre-existing diseases, and the pharmacological therapy for PE.

**Table 1:** Characteristics of the ART patients’ cohort.

	Sp CTRL n=14	OD n=18	HOM n=16
**age (y)**	37 (± 3)	43 (± 4)*	38 (± 3)
***nullipara***	6/14	18/18	11/16
***primigravida***	5/14	9/18	9/16
**BMI (Kg/m^2^)**	20.8 (± 2)	23.4 (± 8)	20.6 (± 3)
**gestation weeks**	40 (± 1)	39 (± 2)	39 (± 2)
**newborn weight (g)**	3353 (± 240)	3155 (± 380)	3266 (± 485)
**placental weight (g)**	560 (± 48)	515 (± 57)*	540 (± 92)
**delivery**			
cesarean	3/14	7/18	5/16
vaginal	11/14	9/18	10/16
**pregnancy complications**	0/14	13/18	6/16
GD	0/14	6/18	1/16
hypertension	0/14	2/18	2/16
pre-eclampsia	0/14	0/18	1/16
COVID-19	0/14	1/18	1/16
**pre-existing diseases**	0/14	11/18	4/16
hypertension	0/14	2/18	0/16
obesity	0/14	1/18	0/16
hypothyroidism	0/14	4/18	2/16
others	0/14	4/18	2/16
**treatments**	0/14	18/18	7/16
LDA	0/14	14/18	3/16
levothyroxine	0/14	5/18	3/16
heparin	0/14	2/18	0/16
niphedipin	0/14	2/18	1/16

Data are expressed as mean ± standard deviation. Asterisks indicate statistical significance (p<0.05) as compared to Sp CTRL. GD, gestational diabetes; LDA, low dose aspirin.

The non-pregnant group (NP) consisted of healthy fertile women (n = 6; age: 35 ± 5 years).

For all cohorts, PE was defined as hypertension arisen suddenly after the 20^th^ week of pregnancy (systolic blood pressure ≥ 140 mmHg and/or diastolic blood pressure ≥ 90 mmHg) with associated proteinuria, greater than or equal to 300 mg/24 hours, often corresponding to 30 mg/dL (1+) in a single sample.

The study was reviewed and approved by the Regional Ethical Committee of FVG (CEUR), Udine, 353 Italy (CEUR-2020-Os-156; Prot. 0022668/P/GEN/ARCS). All participants gave informed, signed consent.

For the characterization of C1q and IgG on STBM, microvesicles derived by dual-placental perfusion of PE and healthy placentae were obtained from the Nuffield Department of Obstetrics and Gynaecology (NDOG), University of Oxford (United Kingdom), as previously described (17). To assess C1q and anti-C1q in the first trimester of PE patients, we used a bank of sera collected for prospective studies from pregnant women followed by the Prenatal Diagnosis and Gynaecologic Unit of the Institute for Maternal and Child Health of IRCSS “Burlo Garofolo” (Trieste, Italy), as previously described (17).

### Collection of data, serum samples and placental tissues

Following enrolment, general clinical characteristics were collected from the patients, including maternal age, pre-gestational BMI, gravidity and parity, smoking habits, pre-conception comorbidities (such as chronic hypertension, diabetes mellitus, endocrinopathies), pre-conception diseases and current medications. Information on ART treatment was retrieved from the clinic’s electronic patient records system (FertiLab Manager), including: the indication for ART treatment, the medication regimen used for the treatment, the treatment’s outcome (endometrium thickness, oestradiol and progesterone levels), data on the number of oocytes retrieved at the time of aspiration, sperm source, fertilization method (IVF or IntraCytoplasmic Sperm Injection, ICSI), transfer of fresh or frozen embryos, and the number of embryos transferred. During pregnancy, relevant obstetric information was gathered from the patient, including Bi-test results, TSH levels during the first trimester, obstetric complications (such as gestational hypertension, PE, gestational diabetes mellitus, cholestasis, dysthyroidism, placental abnormalities), and prescription taken during pregnancy, especially with respect to acetylsalicylic acid. After birth, data on the type of delivery (vaginal delivery, induction of labor, caesarean section), gestational age at birth, gender and weight of the newborn, placental weight, and peripartum maternal and foetal complications were collected.

From each enrolled patient, serum samples and term placental tissue were collected. Blood samples were collected three times, one sample during each trimester of pregnancy; for the groups I, II and III: the first blood sample was collected in conjunction with the blood sampling for the B-test (11-12^th^ week of gestation), the second following the morphological ultrasound scanning (20^th^ week of gestation), and the third following the growth monitoring ultrasound scanning (30^th^ week of gestation). Regarding the IV group, a blood sample was collected upon PE diagnosis. Blood samples were centrifuged at 250 x g for 7 min and serum was stored at -80°C.

After delivery, a cotyledon sample was collected from placenta and fixed in 10% buffered formalin for histological investigations.

### Quantification of CIC-C1q, C1q, and anti-C1q by enzyme-linked immunosorbent assay (ELISA)

Commercial ELISA kits were used to quantify C1q (Hycult Biotech, Cat# HK356-02), anti-C1q (BÜHLMANN, Cat# EK-AC1QA) and C1q bound-circulating immune-complexes (CIC-C1q) (BÜHLMANN, Cat# EK-CIC) in serum samples, following the instructions provided by the manufacturer. The absorbance was read by PowerWave X Microplate Reader (Bio-Tek Instruments) spectrophotometer. After solubilizing microvesicles in Tris buffer saline pH 8, NP40 0.5%, BSA 1 mg/mL, PMSF 1 mol/L (TNNB), C1q and IgG bound to SBTM were analyzed *via* sandwich ELISA, as previously described (12).

### Evaluation of autoantibodies against C1q globular head or collagen-like region by ELISA

The recombinant globular head regions of the human C1q A, B, and C chains (ghA, ghB, and ghC, respectively) were produced as fusion proteins linked to maltose-binding protein (MBP) in *Escherichia coli* BL21 and purified, as described previously (22). The collagen like-regions (CLR) were obtained as described by Defendi et al. (23). Microtiter wells were coated with 4 μg/well of ghA, ghB or ghC in sodium carbonate/bicarbonate buffer (0.035 M NaHCO_3_, 0.015 M Na_2_CO_3_, pH 9.6) or 1 μg/well of CLR in sodium borate buffer (0.2 M H_3_BO_3_, 75 mM NaCl, pH 8.2) overnight at 4°C. The wells were washed with PBS+0.1% Tween 20 and blocked with 2% (w/v) BSA for 1h at 37°C. Patient sera were incubated at a dilution of 1:25-1:50 in PBS/0.75 M NaCl overnight at 4°C. Anti-C1q were detected using an anti-human IgG-alkaline phosphatase (AP)-conjugate (1:50000, Sigma Merck). The binding of secondary antibodies was revealed using the substrate p-nitro phenyl phosphate (pNPP). The absorbance was read at 405 nm using PowerWave X Microplate Reader (Bio-Tek Instruments).

### Immunohistochemistry (IHC)

Placental tissue samples were fixed in 10% buffered formalin and paraffin embedded. Sections were deparaffinized with xylene and rehydrated with decreasing concentrations of ethanol (100%, 95%, 70%) and dH_2_O. Antigen retrieval was performed in Tris-HCl/EDTA buffer, pH 9.0, for 20 min at 95°C. Neutralization of the endogenous peroxidases was performed by adding H_2_O_2_ for 5 min and blocking of non-specific binding was obtained with PBS+2% w/v BSA for 30 min. Samples were incubated first with anti-human C1q polyclonal antibodies (1:500, Dako) overnight at 4°C, and then with anti-rabbit horseradish peroxidase (HRP)-conjugated secondary antibody (1:500, Millipore) for 30 min at room temperature. Staining was revealed by 3-amino-9-ethylcarbazole (AEC) chromogen substrate (Vector Laboratories). Sections were counterstained with Mayer haematoxylin (DiaPath) and examined under a Leica DM 2000 optical microscope. Images were acquired using a Leica DFC 7000 T digital camera (Leica Microsystems, Wetzlar, Germany).

### Evaluation of complement activation

Patients’ sera presenting the highest titers of anti-C1q were added to microtitre wells previously coated with ghA, ghB or CLR and blocked (2.4). To assess the ability of anti-C1q to activate C, wells were then incubated with AB Rh+ pooled sera (1:100 in PBS+2% w/v BSA+0.7mM Ca^++^Mg^++^) for 30 min at 37°C and washed with PBS+0.1% Tween 20. The deposition of C3 was detected using goat anti-human C3 polyclonal antibody (1:5000, Quidel) for 1h at 37°C and anti-goat IgG AP-conjugated (1:30000, Sigma-Aldrich) secondary antibody. The binding was revealed with pNPP and the absorbance was read at 405 nm by PowerWave X Microplate Reader (Bio-Tek Instruments). The mean value obtained by the incubation of ghA, ghB or CLR with pooled AB Rh+ alone was subtracted by patient values.

### Statistical analysis

Mean and standard deviations were calculated for continuous variables, whereas frequencies and percentages were reported for categorical variables. Non-parametric data were assessed by Mann-Whitney U-tests. Patient data were analyzed by t-Student test. Analysis of different groups of data was performed using two-way analysis of variance (ANOVA). Results were expressed as mean ± standard deviations. P-values <0.05 were considered statistically significant. All statistical analyses were performed using GraphPad Prism software 9.0 (GraphPad Software Inc., La Jolla, CA, USA).

## Results

### Evaluation of C1q, CIC and anti-C1q levels in PE and normal pregnancy

**Figure 1:**
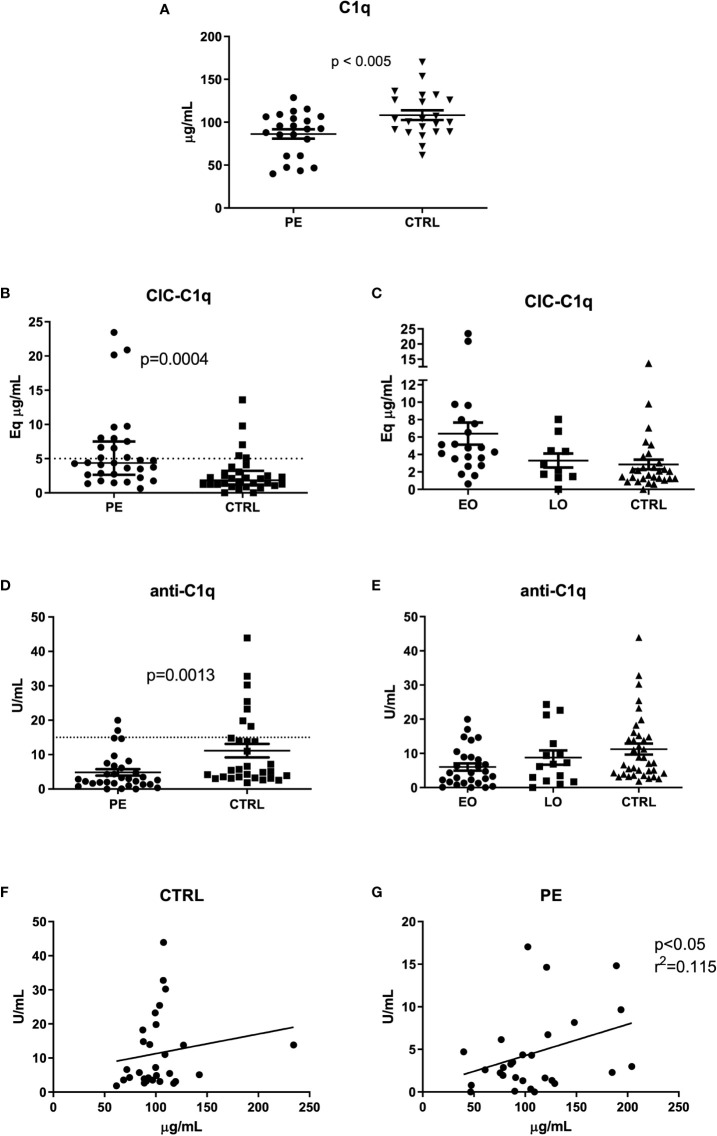
C1q, CIC-C1q and anti-C1q levels at the onset of PE. Measurement of C1q **(A)**, CIC-C1q **(B, C)** and anti-C1q levels **(D, E)** in preeclamptic patients (PE) and healthy pregnant women (CTRL). The PE cohort is further divided into Early-Onset (EO) PE (<34 weeks) and Late-Onset (LO) PE (>34 weeks) patients **(C, E)**. C1q and anti-C1q levels were significantly lower in PE compared to CTRL (p-value calculated using paired T-test, respectively p<0.005 and p=0.0013). Conversely, CIC-C1q levels were significantly higher in the PE group (p=0.0004). The differences were more marked in EO PE. The results are expressed as scattered plots; the line in the middle of the graph represents the mean ± standard error. Anti-C1q and C1q levels were correlated by calculating the coefficient of determination (r^2^) in CTRL **(F)** and in PE patients **(G)**.

We initially confirmed the lower levels of circulating C1q in the sera of the cohort of PE patients in comparison with CTRL (**Figure 1A**), as previously described (17). Subsequently, we proceeded with evaluating the amount of CIC-C1q; we detected a higher amount of CIC-C1q in PE patients (p = 0.0004) as compared to CTRL (**Figure 1B**). Interestingly, early-onset (EO) PE patients showed an even increased level of CIC-C1q as compared to the late-onset (LO) PE and CTRL (**Figure 1C**). As the method for the detection of CIC-C1q is not antigen-specific, the immune-complexes could be heterogeneous in terms of type of antigen and immunoglobulin target. In order to search for possible candidates, we investigated the presence of anti-C1q in our cohorts of patients. We found a significant difference between PE and CTRL (p = 0.0013), but in contrast to CIC data and in accordance with C1q data, PE patients were characterized by lower levels of anti-C1q (**Figure 1D**), which were more evident in the case of a severe disease (EO) (**Figure 1E**). Interestingly, we noted a correlation between low levels of C1q and anti-C1q, and vice-versa, only in the PE group, and not in CTRL, indicating a direct association between C1q and anti-C1q in the disease (**Figures 1F, G**).

### Evaluation of C1q and IgG on STBM

**Figure 2:**
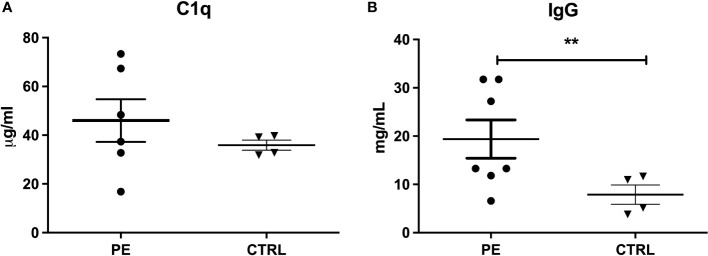
Detection of C1q and IgG on STBM. Analysis of C1q **(A)** and IgG **(B)** bound to STBM was performed by Sandwich ELISA, solubilizing the microvesicles derived from pre-eclamptic (PE, n=7) and control (CTRL, n=4) placentae. No statistical differences were observed between PE and CTRL STBM (Mann-Whitney U-test) as regard to the presence of C1q, whereas greater amount of IgG was found deposited on PE STBM compared to those derived from CTRL placenta. Bars represent mean ± standard error. (**p<0.01, T-test).

Since a significant difference in anti-C1q levels was observed between PE and CTRL sera, we hypothesized its possible binding to placental apoptotic bodies. We investigated the presence of C1q and IgG deposits on microvesicles derived by dual-placental perfusion (STBM), a method previously described by Tannetta et al. (24). We performed a sandwich ELISA on solubilized microvesicles derived from seven PE and four CTRL placentae. As shown in **Figure 2**, C1q and IgG were both present on STBM from PE as well as CTRL placentae, with higher levels in the pathologic ones, despite only IgG showed a significant difference. It is important to note that a higher level of circulating STBM is found in PE patients, which may explain the subtle differences between PE patients and CTRL.

### Evaluation of C1q and anti-C1q levels in sera sample from first trimester

We wanted to investigate if the differences in the levels of C1q and anti-C1q between PE and CTRL were present at an early stage of gestation, and thus, could be used as predictive markers of PE. We quantified anti-C1q in sera collected between the 11^th^ and 13^th^ gestational week in pregnant women who subsequently developed PE. These samples were obtained from a bank of sera collected for prospective studies from pregnant women followed by the Prenatal Diagnosis and Gynaecologic Unit of the Institute for Maternal and Child Health of IRCSS “Burlo Garofolo” (Trieste, Italy), as previously described (17).

**Figure 3:**
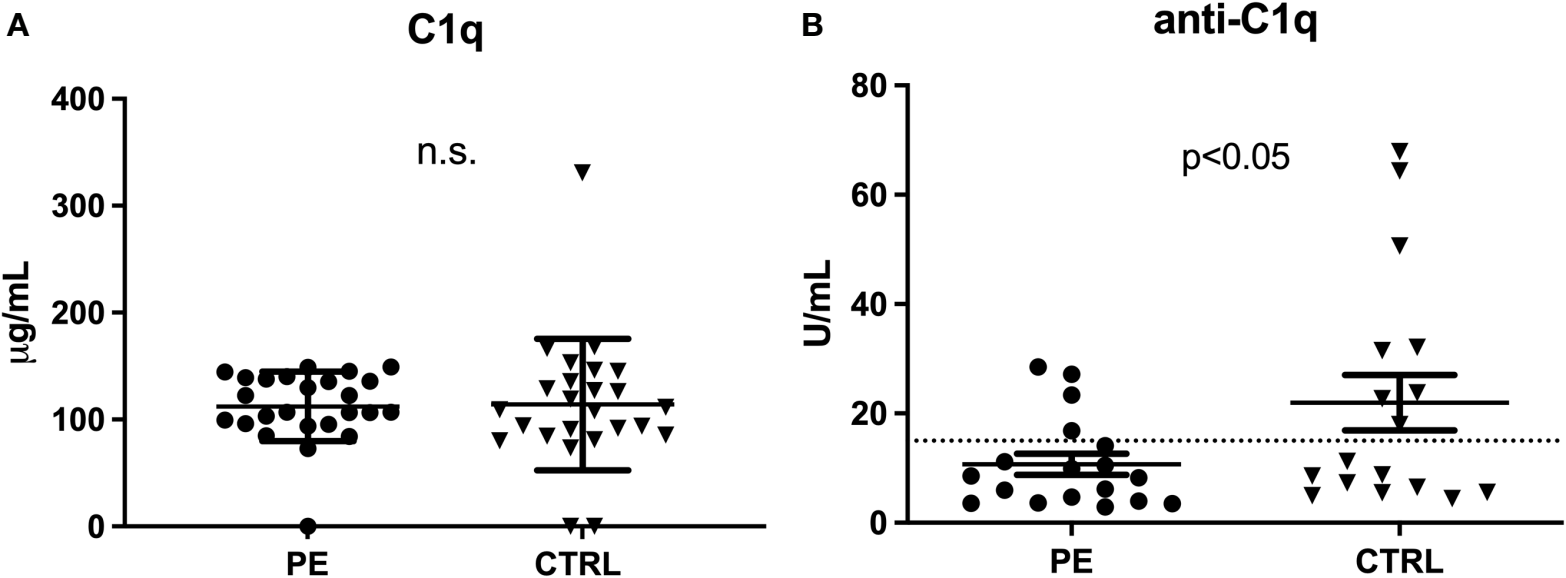
C1q and anti-C1q levels in the first trimester of PE patients. Measurement of C1q **(A)** and anti-C1q **(B)** in first trimester pregnant patients who subsequently developed PE and healthy pregnant women (CTRL). Differences in the C1q levels were not significant (n.s.), while anti-C1q levels were significantly lower in PE compared to CTRL (p<0.05 with paired T-test).

As reported before (17), a difference in C1q levels was not evident in the first trimester of pregnancy (**Figure 3A**). However, significantly lower levels of anti-C1q were present in the first trimester of PE patient samples as compared to CTRL (**Figure 3B**).

### C1q in ART pregnancies

**Figure 4:**
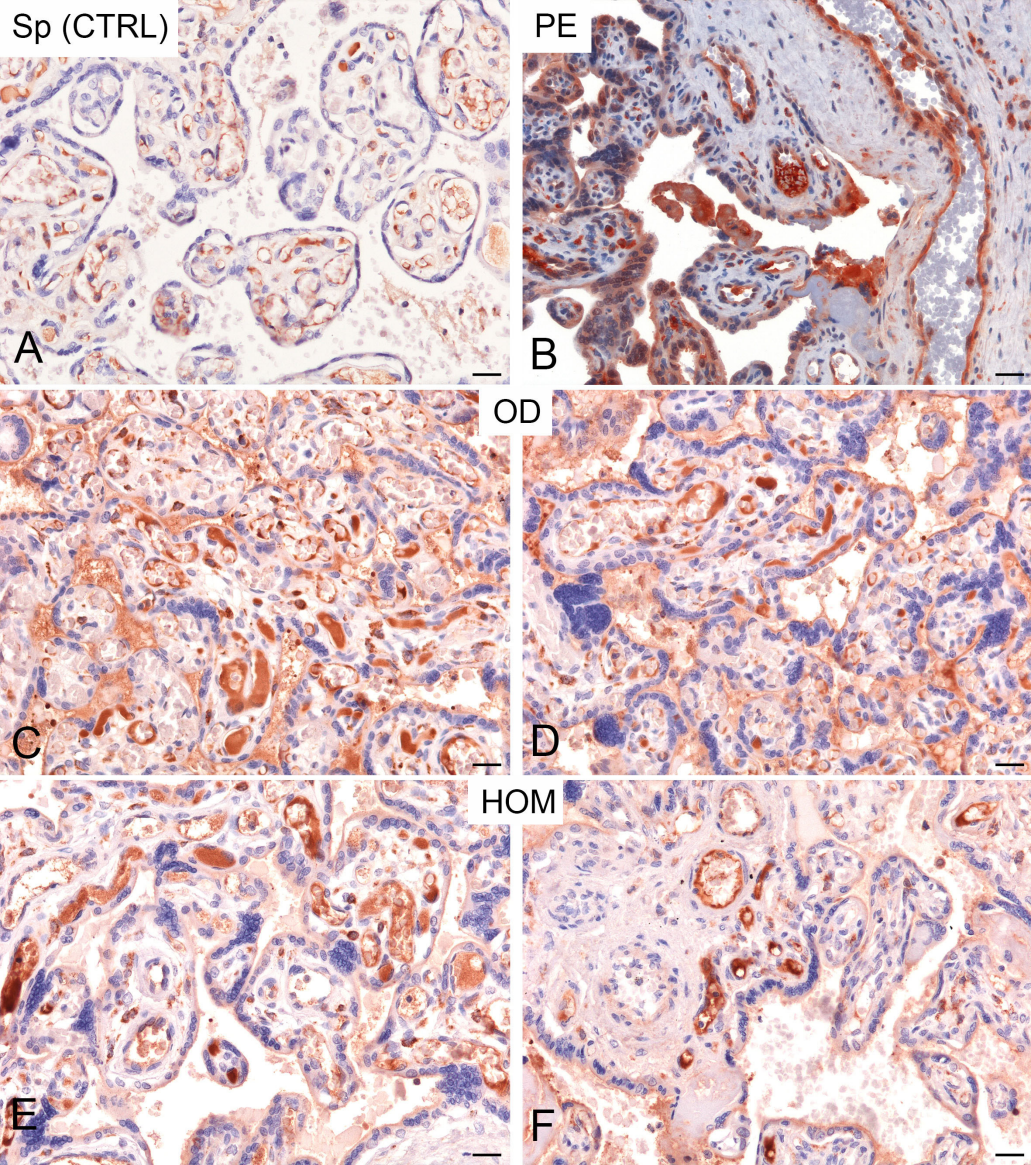
Immunohistochemical analysis of C1q in healthy pregnant women (CTRL) and pre-eclamptic (PE) placentae. Representative microphotographs showing a minimal staining for C1q in placentae derived from spontaneous pregnancies (Sp CTRL) **(A)**. In contrast, higher levels of C1q immunostaining were detected in PE placentae **(B)**. Interestingly, a high variability of C1q expression, but more similar to PE, was observed in placentae derived from oocyte donation pregnancies (OD) **(C, D)** and, less frequently, from homologous IVF pregnancies (HOM) **(E, F)**. AEC (red) chromogen was used to visualize the binding of anti-human C1q antibody. Scale bar 50 µm.

In order to analyse further the role of C1q and anti-C1q in pregnancy, we included, in our study, women undergoing ART. In this case, we distinguished between pregnancies established after OD and homologous-IVF (HOM). Initially, we evaluated the deposition of C1q in paraffin-embedded sections of placentae. We observed higher deposition of C1q in PE patients compared to CTRL; a similar pattern, although slightly variable, was also observed in the placentae derived from OD as well as HOM (**Figure 4**).

### Measurement of C1q and anti-C1q levels throughout pregnancy trimesters

**Figure 5:**
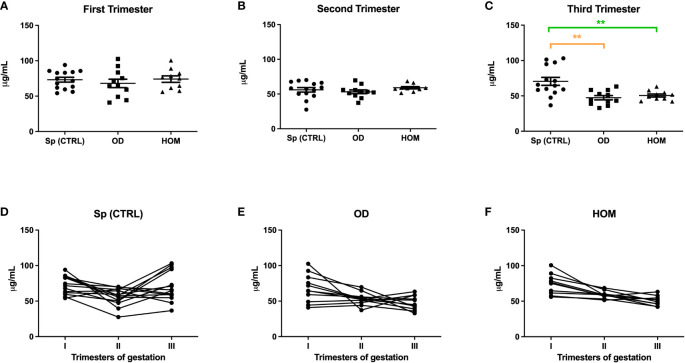
C1q levels during Sp CTRL, OD and HOM pregnancies. Serum levels of C1q were measured in first **(A)**, second **(B)** and third **(C)** trimester, represented as box-plot graphs. No differences were observed in the first and second trimesters, while C1q was significantly lower in the serum of OD and HOM pregnancies in the third trimester compared to Sp CTRL, in a manner consistent with the data previously published for PE patients by Agostinis et al. (17). (**p<0.01, T-test). The before-after plots **(D-F)** describe the changes in C1q levels throughout pregnancy.

To understand the pattern of C1q and anti-C1q throughout gestation, we measured their levels during the three trimesters of pregnancy, by comparing Sp CTRL with ART pregnancies. Circulating levels of C1q observed in the first (**Figure 3****)** and third trimester of PE patients **(** **Figure 1**) were similar to those observed in ART pregnancies: in OD and HOM women, the level of C1q decreased significantly throughout trimesters compared to Sp CTRL. No statistically significant difference was observed in the first and second trimester (**Figure 5**).

**Figure 6:**
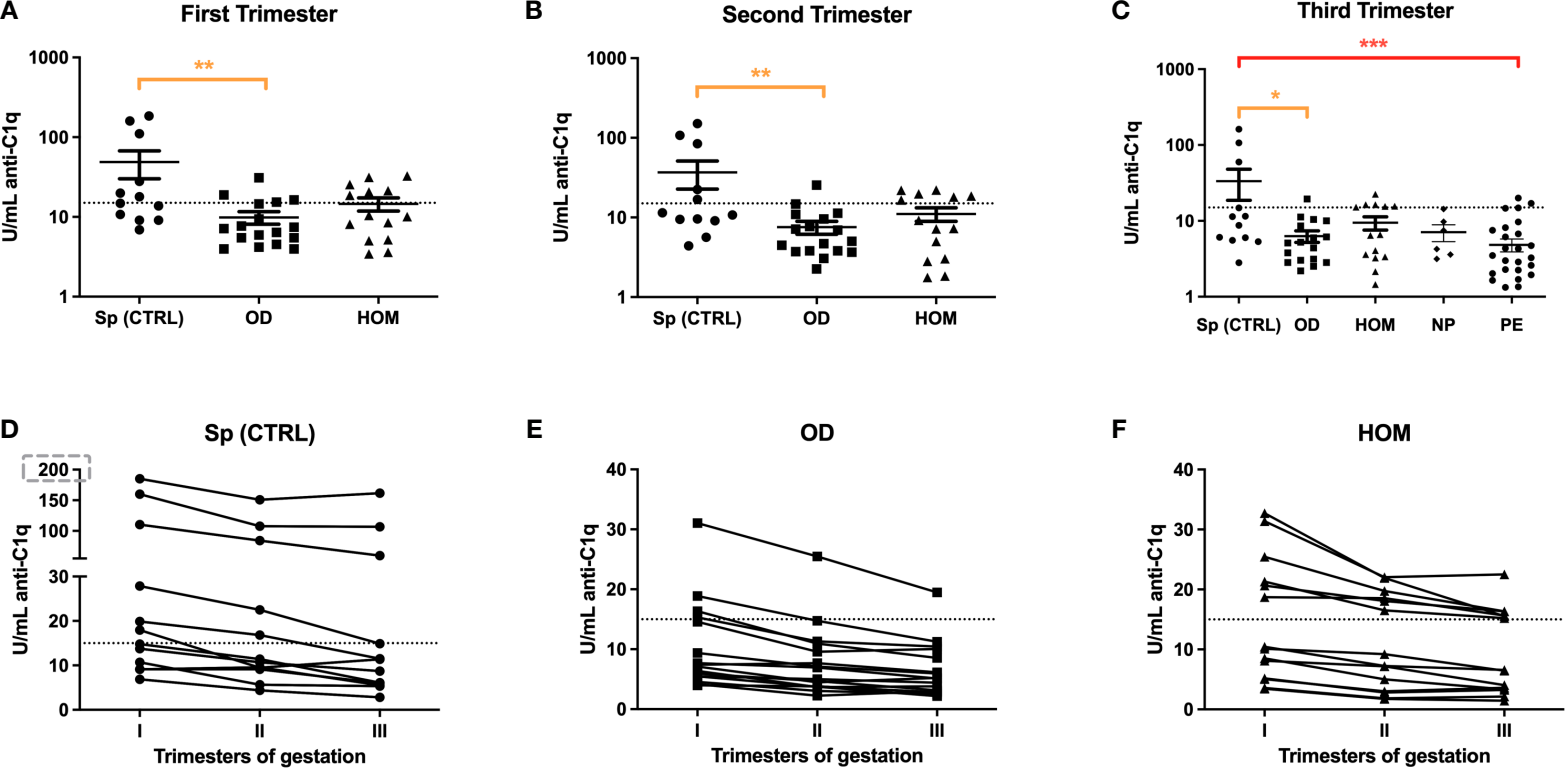
Anti-C1q autoantibodies in Sp CTRL, OD and HOM pregnancies. Serum levels of anti-C1q were measured in the first **(A)**, second **(B)** and third **(C)** trimester of pregnancy. The levels were significantly lower in OD and HOM pregnancies compared to Sp CTRL during all trimesters (*p<0.05; **p<0.01; ***p<0.001; T-test). The before-after plots **(D, E, F)** suggest a decrease in anti-C1q titres with the progression of gestational age.

The same group of patients was then analyzed for anti-C1q level variations during pregnancy. As expected, the trend in ART pregnancies mirrored PE patients. In fact, anti-C1q titres were significantly lower throughout pregnancies in OD and HOM mothers compared to Sp CTRL (**Figure 6**). In the first trimester, the difference was particularly marked: the concentration of anti-C1q ranged between 5 and 100 U/mL in the Sp CTRL, while it was below 10 U/mL in OD and HOM groups. The titre of anti-C1q seems to decrease throughout pregnancy in all groups.

### Evaluation of anti-C1q specificity

**Figure 7:**
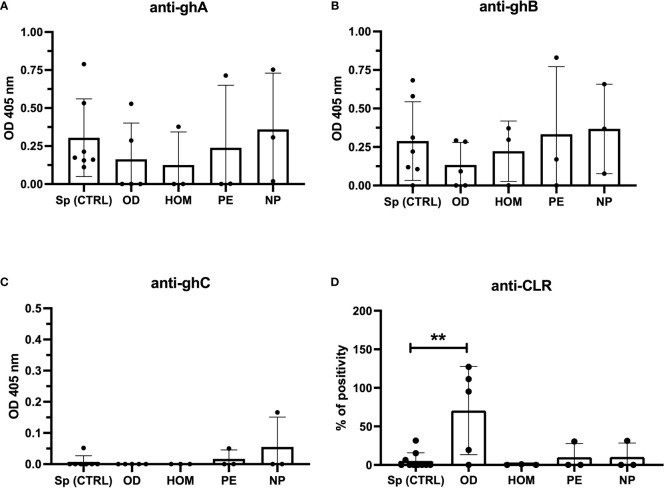
Anti-C1q specific to C1q globular heads and CLR in Sp CTRL, OD, HOM, PE pregnancies and NP women. Anti-C1q were characterized for the specificity of recognized C1q fragments by ELISA. The recombinant globular head regions of the A, B, and C chains (ghA, ghB, and ghC, respectively) **(A–C)** and the CLR **(D)** were coated on microtiter wells. Patient sera were incubated at a dilution of 1:25-1:50 and the detection of anti-C1q antibodies was performed using an anti-human IgG AP-conjugate. Only sera showing anti-C1q levels higher than the cut-off were tested. OD subjects contained higher levels of anti-CLR compared to Sp pregnant women. (**p < 0.01, T-test).

Since Stoyanova et al. reported a different specificity of anti-C1q between healthy and adversely pregnant women (25), we examined the specificity of anti-C1q from the sera used in our study towards C1q globular head modules (ghA, gh and, ghC) and CLR. In particular, we focused our analysis on the sera of the first trimester which previously yielded above the cut-off of 15 U/mL for anti-C1q. As shown in **Figure 7**, even if positive, the OD and HOM groups revealed weak reactivity to ghA and ghB compared to control groups (Sp CTRL and NP). No group showed antibodies against the ghC module. Interestingly, only OD patients had antibodies to CLR, with a very high percentage of positivity found using positive control systemic lupus erythematosus (SLE) patient serum.

Antibodies directed against different modules of C1q were also evaluated for their ability to activate the early steps of the classical pathway *in vitro*. A 96-well plate was coated with ghA, ghB or CLR; after the binding of the autoantibodies, we evaluated the C3 deposition by incubating with a pool of AB+ sera as C source. No correlation between observed Ig levels and C3 deposition or C activation and pathological aspects was clearly revealed (**Supplementary Figure 1**).

## Discussion

C1q, the first recognition subcomponent of the C classical pathway, is a functionally versatile protein; its central role in normal and adverse human pregnancies has been extensively investigated (12, 13, 15, 17, 26). We and others have demonstrated a marked decrease in circulating C1q levels in the sera of PE women (27). In this study, we examined possible reasons for the differences in C1q levels between healthy and PE pregnancies, focusing especially on a potential involvement of anti-C1q.

To further clarify the involvement of C1q in PE pathogenesis, we initially analyzed the level of CIC-C1q in CTRL and PE sera. High levels of CICs in PE patients have been reported (28, 29), which is validated by our current data that PE patients had approximately twice the levels of CIC-C1q compared to CTRL. We also subgrouped the PE cohort in to EO PE and LO PE patients to identify a correlation between CIC-C1q levels and severity of the disease. We found higher levels of immune complexes in the EO PE group. To determine the specificity of CIC and reduction of circulating C1q levels in PE, we turned our attention to anti-C1q. Similar to C1q level, but in contrast to CIC, we found a significantly lower concentration of anti-C1q in PE pregnancies as compared to CTRL (p = 0.0013), especially in the more severe disease (EO). Our results partly contradict a recent study published by Jan Dijkstra and colleagues (30) that evaluated anti-C1q in maternal sera from women with PE and normal pregnancies using samples from 3 cohorts of patients, collected in the Netherlands, Finland and Norway. Their results showed lower anti-C1q levels only in the Netherlands cohort. The discordance of results is probably due to the evaluation in the third trimester of pregnancy, the period in which we observed fewer differences in anti-C1q.

Since one of the main functions of C1q is to mediate apoptotic body clearance, we hypothesized the binding of C1q and subsequently anti-C1q to apoptotic blebs derived from PE placentae. Large quantities of STBM and apoptotic bodies are known to be released in the hypoxic microenvironment of PE placenta in comparison with normal placentae (26, 31). Anti-C1q could have a role in clearing the apoptotic bodies/STBM bound to C1q from the circulation (32, 33). Thus, we looked for C1q and IgG deposition on microvesicles derived by dual-placental perfusion both from PE and CTRL placentae. Higher levels of C1q and IgG, although not statistically significant, were found on PE STBM, consistent with the higher level of STBM circulating in PE compared to normal pregnant women (31).

It is commonly recognized that the pathogenesis of PE starts during the first trimester of pregnancy, due to dysfunctional placental development. However, the onset of clinical signs and symptoms occurs only in the third trimester, making it challenging to identify early biomarkers. Thus, we evaluated the serum levels of C1q and anti-C1q in PE patients and CTRL in the first trimester of pregnancy; a significant reduction of anti-C1q levels was found, suggesting a predictive value to this biomarker. The titre of anti-C1q could reflect the abnormal placentation and subsequent apoptosis occurring in the placenta in the first trimester.

Since women undergoing ART have an increased risk of developing PE, particularly in OD pregnancies (3), we measured the levels of C1q and the novel possible biomarker anti-C1q throughout pregnancy in a longitudinal study, collecting sera along the three trimesters of pregnancies established after OD and homologous-IVF (HOM). Interestingly, in ART pregnancies, the trend of C1q and anti-C1q levels were similar to PE patients, even if these women did not subsequently develop the PE syndrome. This suggests an immunological dysfunction at the foetal-maternal interface in ART pregnancies, which is likely kept under control by low dose aspirin treatment. This hypothesis is supported by the observation of C1q deposition in placentae derived from OD fertilization, comparable to PE where an enhanced C1q deposition could be due to an increased apoptotic incidence, and thus, explain decreased circulating C1q levels.

Since in SLE with lupus nephritis, anti-C1q are mostly targeted against neoepitopes within the collagen-like region of C1q (19, 23, 34), we sought to characterize the C1q domains/modules recognized by the anti-C1q in the current study cohort. The CLR of C1q were first described as the main binding site of anti-C1q (35), but subsequently autoantibodies recognizing the C1q globular regions were also reported later (36, 37). Anti-C1q were found to target a cryptic epitope in the CLR unmasked only after C1q binding to immune-complexes or membranes, but not in circulating C1q (37). We found that the anti-C1q positive sera belonging to OD group showed weak reactivity towards ghA and ghB modules compared to control groups (Sp CTRL and NP). However, these sera contained anti-CLR antibodies, with a very high percentage of positivity calculated on the basis of positive control results (SLE patient sera). Contrary to the results reported by Stoyanova and co-workers (25), we failed to detect anti-CLR antibodies in healthy mothers, where the physiologic pregnancies were characterized by high levels of anti-ghA and anti-ghB. Furthermore, we found that the physiologic anti-C1q directed against gC1q domain were not C-fixing, probably being IgG4 isotype.

The worldwide incidence of PE and its serious impact on the health of the mother and the baby remain disconcerting (2) and there is an urgent need for identifying predictive biomarkers that can be useful in ART pregnancies as an additional screening tool for a more successful management of these patients. Our findings linking anti-C1q levels with adverse pregnancies highlight their potential importance in the early detection of PE pregnancies.

## Conclusions

This study sheds light on several aspects: I) anti-C1q are present at higher levels during healthy pregnancies than in the non-pregnant population, and the levels drop over the three trimesters; II) anti-C1q levels are significantly lower in PE patients since the beginning of the pregnancy; III) OD pregnancies have a lower level of anti-C1q than Sp CTRL; however, when anti-C1q are detected, they exhibit binding specificity towards CLR; IV) anti-C1q in healthy pregnant women are not C-fixing. It is likely that anti-C1q have a physiological role in pregnancy, probably connected to placental microvesicles clearance and inhibition of C activation. Furthermore, ART pregnancies, despite of not displaying symptoms of gestational dysfunction, presented altered levels of C1q and anti-C1q compared to Sp CTRL and similar to PE patients, indicating an immunological dysfunction at the fetus-maternal interface.

## Data availability statement

The original contributions presented in the study are included in the article/**Supplementary Material**. Further inquiries can be directed to the corresponding author.

## Ethics statement

The studies involving human participants were reviewed and approved by CEUR-2020-Os-156; Prot. 0022668/P/GEN/ARCS. The patients/participants provided their written informed consent to participate in this study.

## Author contributions

Conceptualization, CA, GZ, MT, and RB; methodology, MT, IP, LS, AB, EC, TSa, and DV; resources, GZ, FR, TSt, FD, TSa, and NS; data curation, CA, GZ, MT, AM, BB, and AB; writing—original draft preparation, CA, AB, MT, UK and EC; writing—review and editing, CA, AB, AM, UK and RB; supervision, NS, GR and RB; funding acquisition, CA, GR and RB. All authors have read and agreed to the published version of the manuscript.

## Funding

This work was supported by the Ministry of Health, Rome - Italy, in collaboration with the Institute for Maternal and Child Health IRCCS Burlo Garofolo, Trieste – Italy (RC24/19 and 47/20 to GR and 40/20 and 09/21 to CA).

## Acknowledgments

We thank Prof. Ian Sargent and his group of Nuffield Department of Obstetrics and Gynaecology, University of Oxford (John Radcliffe Hospital, Oxford, UK) for providing STBM samples.

## Conflict of interest

The authors declare that the research was conducted in the absence of any commercial or financial relationships that could be construed as a potential conflict of interest.

## Publisher’s note

All claims expressed in this article are solely those of the authors and do not necessarily represent those of their affiliated organizations, or those of the publisher, the editors and the reviewers. Any product that may be evaluated in this article, or claim that may be made by its manufacturer, is not guaranteed or endorsed by the publisher.

## References

[1] Chappell LC Cluver CA Kingdom J Tong S. Pre-eclampsia. Lancet (2021) 398(10297):341–54. doi: 10.1016/S0140-6736(20)32335-7 34051884

[2] Abalos E Cuesta C Grosso AL Chou D Say L. Global and regional estimates of preeclampsia and eclampsia: A systematic review. Eur J Obstet Gynecol Reprod Biol (2013) 170(1):1–7. doi: 10.1016/j.ejogrb.2013.05.005 23746796

[3] Berntsen S Larsen EC la Cour Freiesleben N Pinborg A. Pregnancy outcomes following oocyte donation. Best Pract Res Clin Obstet Gynaecol (2021) 70:81–91. doi: 10.1016/j.bpobgyn.2020.07.008 32741624

[4] Adamson GD de Mouzon J Chambers GM Zegers-Hochschild F Mansour R Ishihara O. International committee for monitoring assisted reproductive technology: world report on assisted reproductive technology, 2011. Fertil Steril (2018) 110(6):1067–80. doi: 10.1016/j.fertnstert.2018.06.039 30396551

[5] European IVF-Monitoring Consortium (EIM) for the European Society of Human Reproduction and Embryology (ESHRE) Wyns C De Geyter C Calhaz-Jorge C Kupka MS Motrenko T Smeenk J. ART in Europe, 2017: Results generated from European registries by ESHRE. Hum Reprod Open (2021) 2021(3):hoab026. doi: 10.1093/hropen/hoab026 34377841 PMC8342033

[6] Pijnenborg R Vercruysse L Hanssens M. The uterine spiral arteries in human pregnancy: Facts and controversies. Placenta (2006) 27(9-10):939–58. doi: 10.1016/j.placenta.2005.12.006 16490251

[7] van der Post JA Lok CA Boer K Sturk A Sargent IL Nieuwland R. The functions of microparticles in pre-eclampsia. Semin Thromb Hemost (2011) 37(2):146–52. doi: 10.1055/s-0030-1270342 21370216

[8] Knight M Redman CW Linton EA Sargent IL. Shedding of syncytiotrophoblast microvilli into the maternal circulation in pre-eclamptic pregnancies. Br J Obstet Gynaecol (1998) 105(6):632–40. doi: 10.1111/j.1471-0528.1998.tb10178.x 9647154

[9] Powe CE Levine RJ Karumanchi SA. Preeclampsia, a disease of the maternal endothelium: The role of antiangiogenic factors and implications for later cardiovascular disease. Circulation (2011) 123(24):2856–69. doi: 10.1161/CIRCULATIONAHA.109.853127 PMC3148781 21690502

[10] Martinez-Varea A Pellicer B Perales-Marin A Pellicer A. Relationship between maternal immunological response during pregnancy and onset of preeclampsia. J Immunol Res (2014) 2014:210241. doi: 10.1155/2014/210241 24987708 PMC4060291

[11] Moreno-Sepulveda J Checa MA. Risk of adverse perinatal outcomes after oocyte donation: a systematic review and meta-analysis. J Assist Reprod Genet (2019) 36(10):2017–37. doi: 10.1007/s10815-019-01552-4 PMC6823473 31440959

[12] Bulla R Agostinis C Bossi F Rizzi L Debeus A Tripodo C. Decidual endothelial cells express surface-bound C1q as a molecular bridge between endovascular trophoblast and decidual endothelium. Mol Immunol (2008) 45(9):2629–40. doi: 10.1016/j.molimm.2007.12.025 PMC2632959 18295334

[13] Agostinis C Bulla R Tripodo C Gismondi A Stabile H Bossi F. An alternative role of C1q in cell migration and tissue remodeling: contribution to trophoblast invasion and placental development. J Immunol (2010) 185(7):4420–9. doi: 10.4049/jimmunol.0903215 20810993

[14] Singh J Ahmed A Girardi G. Role of complement component C1q in the onset of preeclampsia in mice. Hypertension (2011) 58(4):716–24. doi: 10.1161/HYPERTENSIONAHA.111.175919 21859968

[15] Belmonte B Mangogna A Gulino A Cancila V Morello G Agostinis C. Distinct roles of classical and lectin pathways of complement in preeclamptic placentae. Front Immunol (2022) 13:882298. doi: 10.3389/fimmu.2022.882298 35711467 PMC9197446

[16] Girardi G. Pravastatin to treat and prevent preeclampsia. preclinical and clinical studies. J Reprod Immunol (2017) 124:15–20. doi: 10.1016/j.jri.2017.09.009 29028516

[17] Agostinis C Stampalija T Tannetta D Loganes C Vecchi Brumatti L De Seta F. Complement component C1q as potential diagnostic but not predictive marker of preeclampsia. Am J Reprod Immunol (2016) 76(6):475–81. doi: 10.1111/aji.12586 27666323

[18] Potlukova E Kralikova P. Complement component c1q and anti-c1q antibodies in theory and in clinical practice. Scand J Immunol (2008) 67(5):423–30. doi: 10.1111/j.1365-3083.2008.02089.x 18363591

[19] Radanova M Vasilev V Mihaylova G Kosturkova M Kishore U Roumenina L. Autoantibodies against complement classical pathway components C1q, C1r, C1s and C1-inh in patients with lupus nephritis. Int J Mol Sci (2022) 23(16):9281. doi: 10.3390/ijms23169281 36012546 PMC9409282

[20] Menzhinskaya IV Van’ko LV Kashentseva MM Kiryushchenkov PA Sukhikh GT. Incidence of autoantibodies to C1Q complement component in women with miscarriages and autoantibodies to phospholipids and chorionic gonadotropin. Bull Exp Biol Med (2015) 160(2):260–3. doi: 10.1007/s10517-015-3144-x 26639463

[21] Ohmura K Oku K Kitaori T Amengual O Hisada R Kanda M. Pathogenic roles of anti-C1q antibodies in recurrent pregnancy loss. Clin Immunol (2019) 203:37–44. doi: 10.1016/j.clim.2019.04.005 30974291

[22] Kishore U Gupta SK Perdikoulis MV Kojouharova MS Urban BC Reid KB. Modular organization of the carboxyl-terminal, globular head region of human C1q a, b, and c chains. J Immunol (2003) 171(2):812–20. doi: 10.4049/jimmunol.171.2.812 12847249

[23] Defendi F Thielens NM Clavarino G Cesbron JY Dumestre-Perard C. The immunopathology of complement proteins and innate immunity in autoimmune disease. Clin Rev Allergy Immunol (2020) 58(2):229–51. doi: 10.1007/s12016-019-08774-5 31834594

[24] Tannetta DS Dragovic RA Gardiner C Redman CW Sargent IL. Characterisation of syncytiotrophoblast vesicles in normal pregnancy and pre-eclampsia: expression of flt-1 and endoglin. PloS One (2013) 8(2):e56754. doi: 10.1371/journal.pone.0056754 23437230 PMC3577732

[25] Stoyanova V Petrova S Tchorbadjieva M Deliyska B Vasilev V Tsacheva I. New insight into the autoimmunogenicity of the complement protein C1q. Mol Immunol (2011) 48(4):678–82. doi: 10.1016/j.molimm.2010.11.010 21159384

[26] Agostinis C Tedesco F Bulla R. Alternative functions of the complement protein C1q at embryo implantation site. J Reprod Immunol (2017) 119:74–80. doi: 10.1016/j.jri.2016.09.001 27687635

[27] Jia K Ma L Wu S Yang W. Serum levels of complement factors C1q, bb, and h in normal pregnancy and severe pre-eclampsia. Med Sci Monit (2019) 25:7087–93. doi: 10.12659/MSM.915777 PMC6767947 31541546

[28] Stirrat GM Redman CW Levinsky RJ. Circulating immune complexes in pre-eclampsia. Br Med J (1978) 1(6125):1450–1. doi: 10.1136/bmj.1.6125.1450 PMC1604933 647331

[29] Schena FP Manno C Selvaggi L Loverro G Bettocchi S Bonomo L. Behaviour of immune complexes and the complement system in normal pregnancy and pre-eclampsia. J Clin Lab Immunol (1982) 7(1):21–6. 7069775

[30] Dijkstra DJ Lokki AI Gierman LM Borggreven NV van der Keur C Eikmans M. Circulating levels of anti-C1q and anti-factor h autoantibodies and their targets in normal pregnancy and preeclampsia. Front Immunol (2022) 13:842451. doi: 10.3389/fimmu.2022.842451 35432365 PMC9009242

[31] Tannetta D Masliukaite I Vatish M Redman C Sargent I. Update of syncytiotrophoblast derived extracellular vesicles in normal pregnancy and preeclampsia. J Reprod Immunol (2017) 119:98–106. doi: 10.1016/j.jri.2016.08.008 27613663

[32] Huppertz B. IFPA award in placentology lecture: Biology of the placental syncytiotrophoblast–myths and facts. Placenta (2010) 31 Suppl:S75–81. doi: 10.1016/j.placenta.2009.12.001 20042237

[33] Karasu E Eisenhardt SU Harant J Huber-Lang M. Extracellular vesicles: Packages sent with complement. Front Immunol (2018) 9:721. doi: 10.3389/fimmu.2018.00721 29696020 PMC5904200

[34] Pang Y Yang XW Song Y Yu F Zhao MH. Anti-C1q autoantibodies from active lupus nephritis patients could inhibit the clearance of apoptotic cells and complement classical pathway activation mediated by C1q *in vitro* . Immunobiology (2014) 219(12):980–9. doi: 10.1016/j.imbio.2014.07.004 25092568

[35] Uwatoko S Aotsuka S Okawa M Egusa Y Yokohari R Aizawa C. C1q solid-phase radioimmunoassay: Evidence for detection of antibody directed against the collagen-like region of C1q in sera from patients with systemic lupus erythematosus. Clin Exp Immunol (1987) 69(1):98–106. 3498589 PMC1542256

[36] Tsacheva I Radanova M Todorova N Argirova T Kishore U. Detection of autoantibodies against the globular domain of human C1q in the sera of systemic lupus erythematosus patients. Mol Immunol (2007) 44(8):2147–51. doi: 10.1016/j.molimm.2006.09.009 17049989

[37] Stoyanova V Tchorbadjieva M Deliyska B Vasilev V Tsacheva I. Biochemical analysis of the epitope specificities of anti-C1q autoantibodies accompanying human lupus nephritis reveals them as a dynamic population in the course of the disease. Immunol Lett (2012) 148(1):69–76. doi: 10.1016/j.imlet.2012.08.007 22981967

