# Supplementary material for: A longitudinal study of C1q and anti-C1q autoantibodies in homologous and heterologous pregnancies for predicting pre-eclampsia
Source: Front Immunol. 2022 Nov 9;13:1037191. doi: 10.3389/fimmu.2022.1037191 (PMC9682096; doi:10.3389/fimmu.2022.1037191)
Supplement: Supplementary file 1 [file DataSheet_1.docx]

Supplementary Material to

**A longitudinal study of C1q and anti-C1q autoantibodies in homologous and heterologous pregnancies for predicting pre-eclampsia**

**Chiara Agostinis^1$^, Gabriella Zito^1$^, Miriam Toffoli^1^, Isabel Peterlunger^2^, Livia Simoni^1^, Andrea Balduit^1*^, Erica Curtolo^2^, Alessandro Mangogna^1^, Beatrice Belmonte^3^, Davide Vacca^3^, Federico Romano^1^, Tamara Stampalija^1,2^, Tiziana Salviato^4^, Federica Defendi^5^, Nicoletta Di Simone^6,7^, Uday Kishore^8^, Giuseppe Ricci^1,2^ and Roberta Bulla^9^**

^1^Institute for Maternal and Child Health, IRCCS (Istituto di Ricovero e Cura a Carattere Scientifico) Burlo Garofolo, Trieste, Italy

^2^Department of Medical, Surgical and Health Science, University of Trieste, Trieste, Italy

^3^Tumor Immunology Unit, Department of Health Sciences, University of Palermo, Palermo, Italy

^4^Institute of Pathology, University of Modena and Reggio Emilia, Modena, Italy

^5^Laboratory of Immunology, Institute of Biology and Pathology, Centre Hospitalier Universitaire Grenoble Alpes, La Tronche, France

^6^Department of Biomedical Sciences, Humanitas University, Pieve Emanuele, Milan, Italy

^7^IRCCS Humanitas Research Hospital, Rozzano, Milan, Italy

^8^Department of Veterinary Medicine, United Arab Emirates University, Al Ain, United Arab Emirates

^9^Department of Life Sciences, University of Trieste, Trieste, Italy

^$^These authors have contributed equally to this work and share first authorship.

#
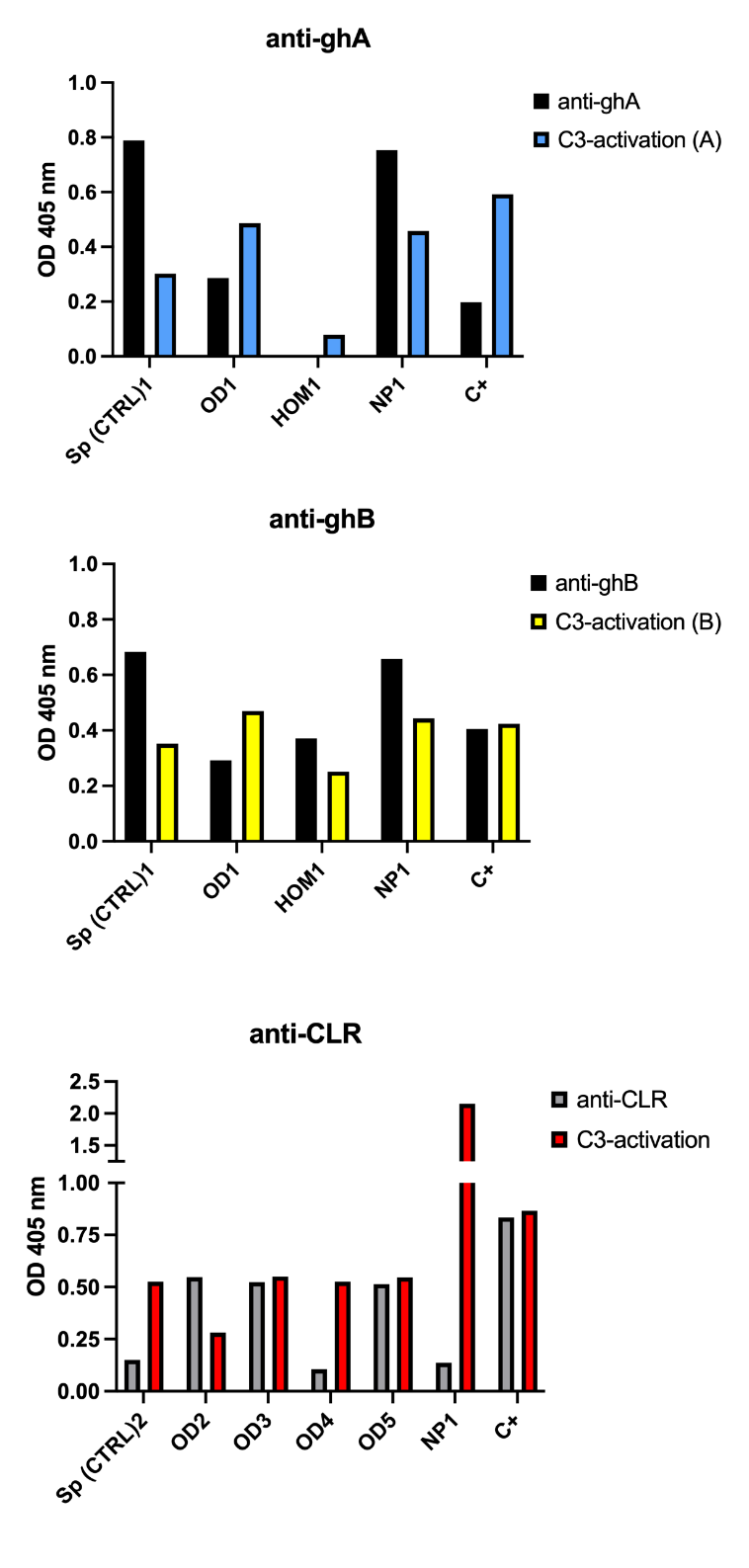
Supplementary Figures

**Supplementary Figure 1.** Evaluation of complement activation. Complement activation by antibodies bound to solid phase-adsorbed ghA, ghB or CLR was evaluated by incubating the fixed autoantibodies with AB Rh+ pooled sera for 30 minutes at 37°C. C3 deposition was detected by specific goat antibodies (Quidel). The AP-conjugated anti-goat IgG (Sigma-Aldrich) was used as secondary antibody.

# Supplementary Tables

**Supplementary Table 1. Table summarizing the characteristics of the three cohorts enrolled in the study.**

|  | Enrolling institutions | Number of patients *vs* control | Gestational age of sample collection | Matching criteria |
| --- | --- | --- | --- | --- |
| 1 | Institute for Maternal and Child Health of the  “Policlinico Gemelli”  (Rome, Italy) | 30 PE  *vs*  30 CTRL | PE diagnosis  (2^nd^/3^rd^ trimester) | age, gestational age, parity |
| 2 | Prenatal Diagnosis and Gynaecologic Unit of the  IRCSS “Burlo Garofolo” (Trieste, Italy) | 20 PE  *vs*  20 CTRL | 1^st^ trimester | age, gestational age, parity |
| 3 | Physiopathology of Human Reproduction and Medically Assisted Procreation Clinic of the IRCSS “Burlo Garofolo” (Trieste, Italy) | 18 OD,  16 HOM  *vs*  14 Sp (CTRL) | longitudinal study (1^st^, 2^nd^ and 3^rd^ trimester) | gestational age |

Abbreviations: BMI, body mass index; CTRL, healthy pregnant women; HOM, homologous *in vitro* fertilization (IVF) pregnancies; OD, oocyte donation; PE, pre-eclamptic patients.

**Supplementary Table 2.** Characteristics of the PE patients’ cohort enrolled at Institute for Maternal and Child Health of the “Policlinico Gemelli” (Rome, Italy).

|  | **PE**  **n=30** |
| --- | --- |
| **gestation weeks** | 33 (±4) |
| **newborn weight (g)** | 1732 (±860) |
| **hypertension** | 75 % |
| **proteinuria (g/L)** | 2.7 (± 4) |
| **IUGR** | 35 % |
| **delivery** |  |
| cesarean | 80 % |
| vaginal | 20 % |
| **pre-existing diseases** |  |
| hypertension | 10% |
| APS | 15% |
| others | 10% |

Data are expressed as mean ± standard deviation and as a percentage. Abbreviations: IUGR, intra uterine growth restriction; APS, antiphospholipid syndrome.
